# Atlastin regulates store-operated calcium entry for nerve growth factor-induced neurite outgrowth

**DOI:** 10.1038/srep43490

**Published:** 2017-02-27

**Authors:** Jing Li, Bing Yan, Hongjiang Si, Xu Peng, Shenyuan L. Zhang, Junjie Hu

**Affiliations:** 1 Department of Genetics and Cell Biology, College of Life Sciences, Nankai University, and Tianjin Key Laboratory of Protein Sciences, Tianjin 300071, China; 2 National Laboratory of Biomacromolecules, Institute of Biophysics, Chinese Academy of Sciences, Beijing 100101, China; 3 Department of Medical Physiology, College of Medicine, Texas A&M Health Science Center, Temple, Texas, USA

## Abstract

Homotypic membrane fusion of the endoplasmic reticulum (ER) is mediated by a class of dynamin-like GTPases known as atlastin (ATL). Depletion of or mutations in ATL cause an unbranched ER morphology and hereditary spastic paraplegia (HSP), a neurodegenerative disease characterized by axon shortening in corticospinal motor neurons and progressive spasticity of the lower limbs. How ER shaping is linked to neuronal defects is poorly understood. Here, we show that dominant-negative mutants of ATL1 in PC-12 cells inhibit nerve growth factor (NGF)-induced neurite outgrowth. Overexpression of wild-type or mutant ATL1 or depletion of ATLs alters ER morphology and affects store-operated calcium entry (SOCE) by decreasing STIM1 puncta formation near the plasma membrane upon calcium depletion of the ER. In addition, blockage of the STIM1-Orai pathway effectively abolishes neurite outgrowth of PC-12 cells stimulated by NGF. These results suggest that SOCE plays an important role in neuronal regeneration, and mutations in ATL1 may cause HSP, partly by undermining SOCE.

The ER contains two morphological domains with distinct functions^1^,^2^. Cisternal-like sheets are mostly localized in the perinuclear region. The flattened surface of ER sheets allows better docking of translating ribosomes and plays a critical role in protein synthesis. Cylindrical tubules form a reticular network that is most prominent in the cell periphery. The curved membranes of the tubules are proposed to facilitate vesicle formation^3^,^4^,^5^, and the tubular network may be involved in lipid metabolism and membrane contact site formation^6^,^7^,^8^.

The tubules are generated and stabilized by integral membrane proteins, such as reticulons (RTNs) and DP1/Yop1p^9^,^10^. When reconstituted into proteoliposomes, purified Yop1p and Rtn1p can generate tubules *in vitro*^10^; deletion of these proteins causes ER sheet expansion at the cost of the tubules^9^. The network is formed with merging of the tubules by dynamin-like GTPase atlastin (ATL)^11^,^12^,^13^. Purified and reconstituted *Drosophila* ATL can fuse vesicles *in vitro*^12^,^14^, and depletion of ATLs or overexpression of a dominant-negative form results in unbranched ER tubules in mammalian cells^11^,^15^, indicating a lack of fusion.

The integrity of the ER tubular network is important. Deletion of ATL in *Drosophila* causes neuronal defects^16^,^17^, and mutants of plant homolog RHD3 exhibit short and wavy root hairs^18^,^19^,^20^. In human, mutations in ATL1, the dominant isoform in the central nervous system, are linked to hereditary spastic paraplegia (HSP), a neurodegenerative disease characterized by axon shortening in corticospinal motor neurons and progressive spasticity and weakness of the lower limbs^21^,^22^. Thus, ATL1 is also termed SPG3A. Structural and biochemical analysis has confirmed that most ATL1 mutations are defective in fusion, but how altered ER morphology leads to neuronal defects is not clear.

ER tubules have been reported to make direct contact with other membranes, such as mitochondria^23^,^24^,^25^, endosomes^26^,^27^, and plasma membranes (PMs)^28^, mediating organelle fission, transferring lipids, or coordinating calcium signaling. One role of ER-PM contact sites is to facilitate store-operated calcium entry (SOCE). When the calcium stored in the ER is depleted, as often triggered by inositol trisphosphate, ER-localized Ca^2+^ sensor STIM changes conformation, forms oligomers in the proximity of the PM, and activates Ca^2+^ release-activated Ca^2+^ (CRAC) channel Orai on the PM to replenish the Ca^2+^ content^29^,^30^,^31^,^32^,^33^. Defects in ER morphology, as demonstrated in Rtn4-deleted MEF cells, affect SOCE^34^. Replenishing Ca^2+^ by SOCE is critical for T cell activation and many other immune responses^35^,^36^. Consistently, mutations in STIM1 and Orai1 have been shown to cause severe combined immunodeficiency (SCID) in humans^37^,^38^. Whether SOCE plays a role in other physiological systems is yet to be determined.

Here, we elucidate a possible link between ER morphology, SOCE, and neurite outgrowth. We show that defects in ER fusion diminish nerve growth factor (NGF)-induced neurite outgrowth in PC-12 cells. In addition, altered ER morphology decreases STIM1 puncta formation near the PM and Ca^2+^ re-entry, and an altered SOCE pathway affects stimulated neurite outgrowth.

## Results

**Figure 1:**
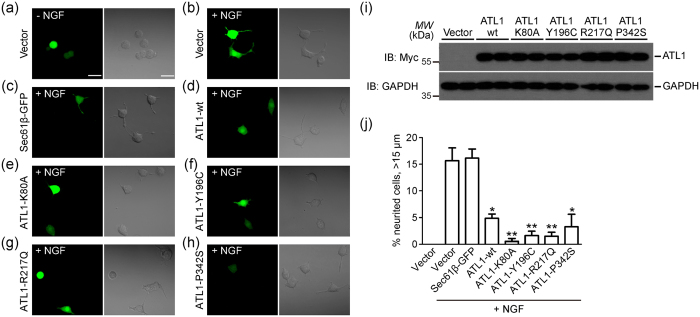
Overexpression of ATL1 impaired neurite outgrowth in PC-12 cells. **(a** and **b)** Representative images for neurite outgrowth in GFP-transfected PC-12 cells with (**b**) or without (**a**) 100 ng/ml NGF treatment for 48 hours. **(c–h)** Images of NGF-treated PC-12 cells transfected with Sec61β-GFP (**c**) or co-transfected with GFP and Myc-ATL1-wt (**d**) Myc-ATL1-K80A (**e**) Myc-ATL1-Y196C (**f**) Myc-ATL1-R217Q (**g**) or Myc-ATL1-P342S (**h**). **(i)** Western blot for Myc-vector, Myc-ATL1-wt, Myc-ATL1-K80A, Myc-ATL1-Y196C, Myc-ATL1-R217Q, and Myc-ATL1-P342S in PC-12 cells. GAPDH was used as a loading control. Full length blot are presented in Supplementary Figure S6a. **(j)** Quantification of the cells with neurites longer than 15 μm. The percentage was determined from three independent assays. (Vector without NGF, n = 62; vector, n = 230; Sec61β-GFP, n = 134; ATL1-wt, n = 134; ATL1-K80A, n = 170; ATL1-Y196C, n = 112; ATL1-217Q, n = 141; ATL1-P342S, n = 165). Scale bar = 20 μm. *P < 0.05; **P < 0.01.

To test whether neurite outgrowth is affected by changes in ER morphology caused by defects in ER fusion, we transfected PC-12 cells with Myc-tagged human ATL1 and performed a neurite outgrowth assay. Given that ATLs form homotypic or heterotypic interactions, overexpression of ATL mutants confers a dominant-negative effect on the tubular ER network^11^. When PC-12 cells were treated with NGF, neurites longer than 15 μm were visualized by the expression of a cytosolic GFP in more than 15% of cells (Fig. 1a,b,j). This outgrowth was strongly inhibited when ATL1 K80A, a GTP binding-defective mutant, was co-expressed (Fig. 1e,i). Similar defects were observed when several HSP-causing ATL1 mutations, including Y196C, R217Q, and P342S, were tested (Fig. 1f–i).

Because PC-12 cells exhibited a less characteristic peripheral tubular ER network, we tested the impacts of these mutants on ER morphology in COS-7 cells. Consistent with previous reports, the overexpression of these ATL1 mutants resulted in long unbranched ER tubules (Figs S1a and S2e) indicative of defects in ER fusion between tubules. Overexpression of wild-type ATL1 also cause aberrant ER (Fig. S2e), in this case sheet expansion due to excess ER fusion. Similar to ATL1 mutants, PC-12 cells transfected with wild-type ATL1 reduced NGF-induced neurite outgrowth (Fig. 1d). In contrast, when Sec61β-GFP (a commonly used ER marker) was overexpressed, no detectable changes in ER morphology were observed (Fig. S1a) and neurite outgrowth was successfully induced by NGF as in control cells (Fig. 1c,j). These results suggest that ATL plays a role, likely through the regulation of ER morphology, in NGF-induced neurite outgrowth.

**Figure 2:**
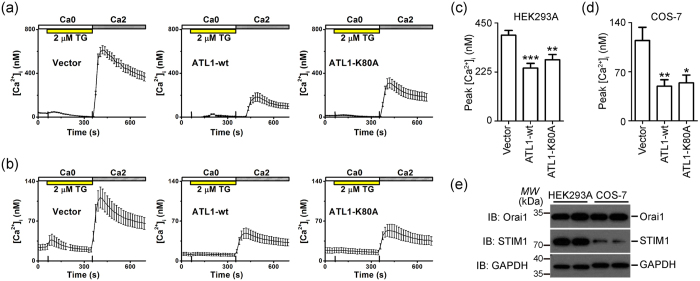
Overexpression of ATL1 reduced TG-evoked SOCE. **(a)** Representative [Ca^2+^]_i_ data illustrating SOCE in HEK293A cells overexpressing GFP + Myc-vector (***left***), GFP + Myc-ATL1-wt (***middle***), or GFP + Myc-ATL1-K80A (***right***) after treatment with 2 μM TG for 5 minutes. **(b)** Representative intracellular free calcium ([Ca^2+^]_i_) recordings showing TG-triggered SOCE in COS-7 cells transfected with GFP + Myc-vector (***left***), GFP + Myc-ATL1-wt (***middle***), or GFP + Myc-ATL1-K80A (***right***). **(c)** Averaged peak values of [Ca^2+^]_i_ were collected from corresponding HEK293A cells (Vector, n = 112; ATL1-wt, n = 43; ATL1-K80A, n = 54). **(d)** Averaged peak values of [Ca^2+^]_i_ were collected from corresponding COS-7 cells (Vector, n = 24; ATL1-wt, n = 29; ATL1-K80A, n = 15). **(e)** Western blot of the endogenous expression levels of STIM1 and Orai1 in HEK293A or COS-7 cells. GAPDH was used as a loading control. Full length blot are presented in Supplementary Figure S6b. *P < 0.05; **P < 0.01; ***P < 0.001.

To probe the intracellular changes caused by defective ATL, we investigated whether ATL is involved in SOCE, a process that relies on ER morphology as suggested previously^34^. First, we measured thapsigargin (TG)-evoked SOCE in HEK293A cells, in which CRAC channel Orai1 and calcium sensor STIM1 are abundantly expressed. Upon overexpression of wild-type ATL1 or K80A, SOCE was reduced in these cells (Fig. 2a,c). However, SOCE remained unchanged when Sec61β-GFP was overexpressed (Fig. S2a,b). Similar results were obtained in COS-7 cells (Fig. 2b,d), in which the ER morphology is easier to analyze. The expression levels of Orai1 in COS-7 cells were comparable to the expression levels in HEK293A cells, but the levels of STIM1 were relatively low (Fig. 2e). As expected, TG-evoked SOCE in COS-7 cells was low, with a peak cytosolic [Ca^2+^] < 140 nM (Fig. 2d), and was still subject to inhibition by ATL1 proteins, including SPG3A mutants Y196C, R217Q, and P342S (Fig. S1b), but not Sec61β-GFP (Figs 2b and S2c,d). Defective SOCE is less likely attributed to changes in the levels of endogenous STIM1 and Orai1 upon ATL1 overexpression (Fig. S2f).

**Figure 3:**
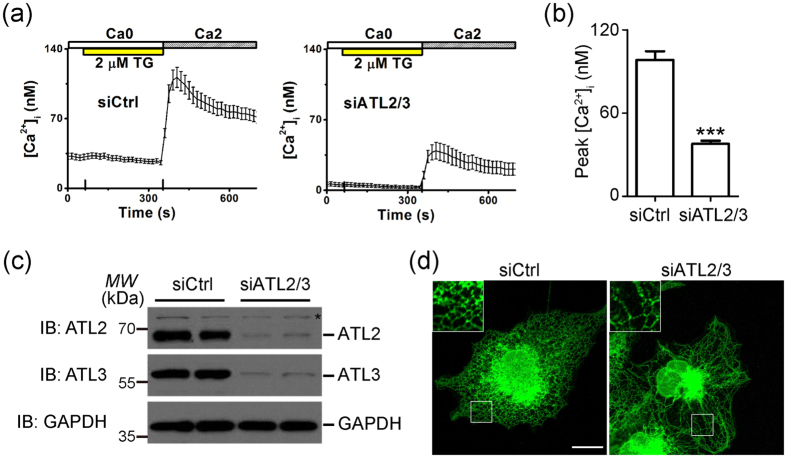
Depletion of ATLs reduced SOCE. **(a)** Representative intracellular free calcium ([Ca^2+^]_i_) recordings showing TG-triggered SOCE in COS-7 cells transfected with control non-targeting siRNA (***left***) or siRNAs target ATL2 and ATL3 (***right***) for 72 h. **(b)** Averaged peak values of [Ca_2+_]_i_ were collected from corresponding COS-7 cells (siCtrl, n = 70; siATL2/3, n = 71). **(c)** ATL2 and ATL3 expression was detected by immunoblotting. An asterisk (*) indicates a non-specific band. Full length blot are presented in Supplementary Figure S6c. **(d)** The ER morphology was visualized by calreticulin antibody staining. Enlarged areas are marked by the small squares, showing normal (***left***) and long, unbranched ER tubular networks (***right***). Scale bar = 15 μm. ***P < 0.001.

To confirm the role of ER morphology in SOCE, we depleted ATL2 and ATL3, the predominant forms of ATL in COS-7 cells, using siRNA and measured the TG-evoked SOCE. Consistent with previous reports^11^,^13^, ATL depletion resulted in similar ER morphology defects as the overexpression of ATL1 mutants (Fig. 3c,d). As predicted, TG treatments induced less SOCE in these cells than in control cells (Fig. 3a,b). Finally, we tested whether SOCE is also linked to ATL in PC-12 cells. PC-12 cells express all three ATLs (Fig. S3c). siRNAs against all rat ATLs were transfected. While ATL2 and 3 were largely depleted, siRNA against rat ATL1 was less efficient (Fig. S3c). As expected, the double knockdown cells exhibited mild decrease in SOCE (Fig. S3a,b), even though less noticeable changes were seen in neurite outgrowth assays (Fig. S3d). Taken together, these results suggest that ATL affects SOCE, likely by regulating ER shape, and Ca^2+^ reentry is sensitive to morphological changes in the ER.

Decreased levels of cytosolic calcium could be explained by either defective calcium influx by Orai channel or inefficient release of Ca^2+^ by IP3 receptors. Because ATP triggers Ca^2+^ release from ER through IP3 receptor, we tested whether ATL overexpression or depletion damages the IP3 receptor by monitoring cytosolic Ca^2+^. No obvious differences were detected between wild type, ATL-overexpressed (Fig. S4a,b), or depleted (Fig. S4c,d) COS-7 cells.

**Figure 4:**
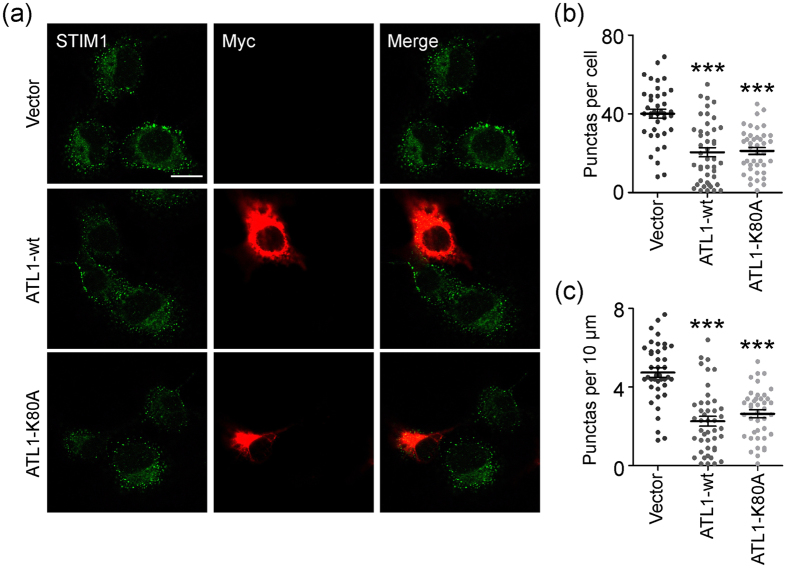
Overexpression of ATL1 inhibited STIM1 puncta formation. **(a)** Endogenous STIM1 puncta (green) and overexpressed Myc-vector, Myc-ATL1-wt, or Myc-ATL1-K80A (red) were stained in COS-7 cells after TG treatment. **(b)** Quantification of STIM1 puncta per cell. **(c)** Quantification of the number of pericellular STIM1 puncta in 10 μm perimeter per cell (Vector, n = 42; ATL1-wt, n = 46; ATL1-K80A, n = 42). Scale bar = 15 μm. ***P < 0.001.

The activation of CRAC channel Orai requires the accumulation of STIM1 at ER-PM contact sites. Thus, we tested whether reduced SOCE upon ATL overexpression is caused by inefficient organization of STIM1 near the PM. We used TG treatments to induce SOCE and monitored STIM1 puncta formation via immunofluorescent staining of endogenous STIM1. In cells transfected with empty vector, TG triggered massive STIM1 puncta formation (Fig. 4a–c). Similarly, STIM1 puncta were greatly induced by TG in cells overexpressing GFP or Sec61β-GFP (Fig. S2g-i). In contrast, puncta formation was reduced in cells transfected with wild-type ATL1 or K80A mutant (Fig. 4a). These results suggest that defects in ER morphology caused by ATL overexpression affects SOCE by interfering with STIM1 puncta formation in the cell cortex.

**Figure 5:**
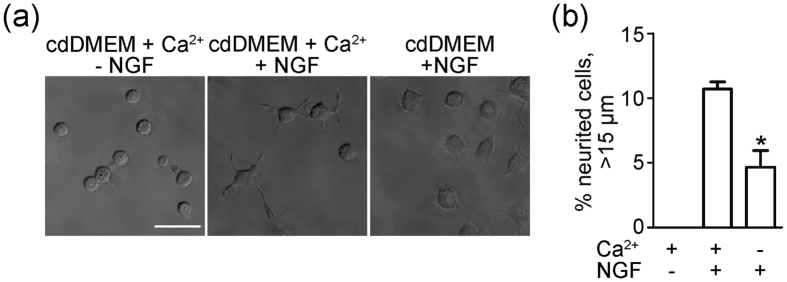
Extracellular Ca^2+^ was essential for NGF-induced neurite outgrowth. **(a)** Representative images for neurite outgrowth in PC-12 cells treated with 2 mM Ca^2+^, NGF + 2 mM Ca^2+^, or NGF only and cultured in calcium-deficient DMEM (cdDMEM). **(b)** The percentage of neurited cells with neurites longer than 15 μm (No NGF, n = 342, NGF + Ca^2+^, n = 374; NGF only, n = 587). *P < 0.05.

**Figure 6:**
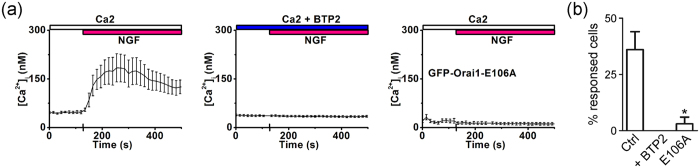
NGF-induced Ca^2+^ mobilization was suppressed by CRAC channel inhibition. **(a)** Representative [Ca^2+^]_i_ response to 2 μg/ml NGF treatment in PC-12 cells (***left***); representative [Ca^2+^]_i_ recording showing NGF-induced [Ca^2+^]_i_ mobilization from PC-12 cells treated with 10 μM BTP2 (***middle***); and representative [Ca^2+^]_i_ data illustrating [Ca^2+^]_i_ mobilization after NGF treatment in PC-12 cells overexpressing GFP-Orai1-E106A (***right***). **(b)** The percentage of cells responsive to NGF was calculated from three independent assays (Ctrl, n = 188; + BTP2, n = 70; E106A, n = 36). *P < 0.05.

To probe the linkage between SOCE and neurite outgrowth, we measured the dependence of neurite outgrowth in PC-12 cells on extracellular calcium. When NGF-activated neurite outgrowth was tested in calcium-deficient DMEM (cdDMEM), the percentage of cells with long neurites (>15 μm) was greatly decreased compared to PC-12 cells supplemented with 2 mM CaCl_2_ (Fig. 5a,b). Consistently, NGF treatment induced Ca^2+^ mobilization in PC-12 cells (Fig. 6a), and approximately 36% of cells responded to treatment with NGF (Fig. 6b). We also tested whether calcium influx during neurite outgrowth is regulated by Orai1 and STIM1 by treating cells with CRAC channel blocker BTP2 or overexpressing Orai1 dominant-negative mutant E106A. NGF-induced Ca^2+^ mobilization was significantly suppressed (Fig. 6a,b). These results suggest that Orai1-STIM1 plays a role in NGF-activated calcium influx.

**Figure 7:**
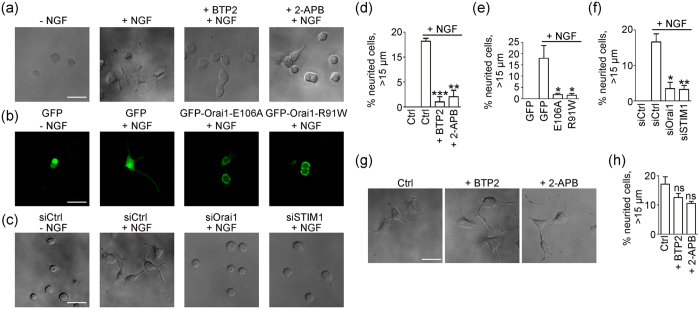
NGF-induced neurite outgrowth was sensitive to CRAC channel inhibition. **(a)** Representative images for neurite outgrowth in PC-12 cells with DMSO, NGF, NGF and 10 μM BTP2, or 50 μM 2-APB treatment for 48 hours. **(b)** Representative images for neurite outgrowth in PC-12 cells transfected with GFP, GFP-Orai1-E106A, or GFP-Orai1-R91W with or without NGF treatment for 48 hours. **(c)** Representative images for neurite outgrowth in PC-12 cells transfected with control siRNA, siOrai1, or siSTIM1 with or without NGF treatment. **(d)** Quantification data for the cells with long neurites in panel (a) (Ctrl without NGF, n = 422; Ctrl, n = 721;+BTP2, n = 982;+2-APB, n = 1031). **(e)** Quantification data for panel (b) (GFP without NGF, n = 40; GFP, n = 106; E106A, n = 298; R91W, n = 334). **(f)** Quantification data for panel (c) (siCtrl without NGF, n = 480; siCtrl, n = 323; siOrai1, n = 287; siSTIM1, n = 401). **(g)** PC-12 cells were treated with NGF for 48 hours, followed by DMSO, 2-APB, or BTP2 treatment for 24 hours. **(h)** Neurite length was quantified 72 hours later (Ctrl, n = 360; +BTP2, n = 375;+2-APB, n = 342). *P < 0.05; **P < 0.01; ***P < 0.001; ns, P ≥ 0.05 compared to control. Scale bar = 20 μm.

We also investigated whether SOCE is critical for NGF-activated neurite outgrowth. NGF-induced neurite outgrowth was inhibited after cells were treated with BTP2 and 2-amino-ethyl diphenylborinate (2-APB) (Fig. 7a,d), which effectively blocked CRAC channels (Fig. S5a–c,j). PC-12 cells transfected with Orai1 dominant-negative mutant Orai1-E106A or Orai1-R91W exhibited suppressed neurite outgrowth (Fig. 7b,e) and a diminished TG-evoked Ca^2+^ influx (Fig. S5d–f,k). Similarly, depletion of rat STIM1 or Orai1 by siRNA caused a failure in stimulated Ca^2+^ mobilization (Fig. S5g–i,l,m) and inhibited neurite outgrowth (Fig. 7c,f).

Finally, to test whether SOCE is involved in the maintenance of grown neurites, we performed post-NGF inhibition of SOCE. PC-12 cells were treated with NGF for 48 hours to induce neurite outgrowth and then treated with DSMO, BTP2, or 2-APB for 12 hours. The percentage of cells with long neurites remained the same (Fig. 7g,h), indicating that grown neurites are not sensitive to CRAC channel inhibition. Taken together, these results suggest that NGF-activated neurite outgrowth in PC-12 cells depends on SOCE.

## Discussion

Our results demonstrate a key role of ATL1 in neurite outgrowth, which is a potential mechanism underlying HSP processes. Overexpression of ATL1 and its GTP-binding mutation K80A suppresses NGF-induced neurite outgrowth in PC-12 cells in a SOCE-dependent manner. Similar to the ATL1-K80A mutant, the SPG3A mutations (Y196C, R217Q, and P342S) exhibit a strong dominant-negative effect^39^. Y196 is positioned close to α2, which consists of the dimer interface of the GTPase domain, and α6, which leads to the linker to 3HB^14^,^40^. Y196C may affect dimerization of or conformational changes in ATL1. R217 is located in the RD motif of the GTPase domain, which engages the ribose of the nucleotide, and substitution by Q results in diminished GTP binding^15^,^40^. The P342S mutation is located in the linker region between the GTPase domain and the 3HB, and plays a key role in conveying a power stroke from the GTPase to the 3HB^41^. These three SPG3A mutants represent different types of defects, but all compromise NGF-induced neurite outgrowth and TG-evoked SOCE.

Overexpression of wild-type ATL1 and SPG3A mutants or ATL knockdown cause dramatic changes in ER morphology, which may affect the formation of ER-PM junctions. Such membrane junctions are critical for SOCE. We found that SOCE was significantly decreased and the number of STIM1 puncta at the ER-PM junctions reduced. Disruption of the tubular ER network may result in a decreased frequency of STIM1 translocation to ER-PM junctions and decreased SOCE. Similarly, deletion of RTN4a, an ER tubule formation protein, causes expansion of ER sheets at the cost of tubules and attenuated SOCE^34^.

The mechanism underlying neurite outgrowth remains unknown. Our studies have demonstrated that SOCE is involved, likely by regulating the cytosolic calcium level. The activation of tyrosine kinase receptor-A (TRKA), the cell surface NGF receptor, is followed by calcium release from the ER through the PLCγ-PIP2-IP3 signaling pathway^42^. ER store depletion induces STIM1/Orail1-mediated SOCE. We showed that the calcium influx mediated by STIM1/Orai1 is crucial for NGF-induced neurite outgrowth, which can be inhibited by overexpression of wild-type ATL1 or the K80A or SPG3A mutant, compromising SOCE. In addition, CRAC channel attenuation suppresses neurite outgrowth. Consistent with our findings, STIM1 has been implicated in growth cone turning focused on axon guidance^43^,^44^. Mutations in human STIM1 and Orai1 have been found in patients with immunodeficiency, highlighting the importance of SOCE in T cell activation. Our studies suggest that SOCE may also be critical for neuronal signaling even though the expression levels of Orai/STIM in the nervous system are relatively low.

Unravelling the abnormal physiological activities in neurons caused by SPG mutants is a critical step to further understanding the pathogenic mechanism of HSP. Defects caused by various SPG mutants are diverse and include mitochondrial transportation^41^,^45^ and lipid droplets in neurons^46^,^47^, but have converging symptoms. Our results provide evidence that ATL1 mutations may not always lead to a distal, dying-back degeneration of axons as previously expected. Defects could occur at the early stage with abnormal neurite development, as observed in a neuronal differentiation assay using HSP patient-derived iPSCs^41^. The complex pathogenic mechanisms of HSP coincide with a broad range of mutated genes associated with the disease.

## Materials and Methods

### Plasmids and reagents

The pGW1-Myc-ATL1-wt and pGW1-Myc-ATL1-K80A plasmids^15^ and GFP-Orai1-E106A and GFP-Orai1–R91W were described previously^48^. The SPG3A mutants, including Myc-ATL1-Y196C, R217Q, and P342S, were created by exchanging the corresponding codons using the QuikChange XL Site-Directed Mutagenesis Kit (Agilent Technologies).

TG, 2-APB, and BTP2 were purchased from VWR. NGF and Fura-2 AM were purchased from Life Technologies. Horse serum and L-glutamine were purchased from Sigma. Fetal bovine serum was purchased from Omega. Rabbit anti-calreticulin, anti-ATL2, and anti-ATL3 antibodies were purchased from Abcam. Mouse anti-Myc antibody was purchased from Santa Cruz Biotechnology. Rabbit anti-Orai1 antibody was purchased from Alomone Labs. Mouse anti-Orai1 was purchased from Abcam. Mouse anti-GAPDH was purchased from Fitzgerald. Rabbit anti-STIM1 antibody was purchased from Cell Signaling. Control non-targeting siRNA, rat STIM1 siRNA, and rat Orai1 siRNA, which includes four different siRNA oligonucleotides, were purchased from Dharmacon. ATL2 and ATL3 siRNAs used in COS-7 cells were synthesized as described previously^15^. The cdDMEM was bought from GE. Ca0 and Ca2 solutions were prepared as described previously^48^.

### Cell culture and transfection

Human embryonic kidney (HEK) 293 A cells (Invitrogen) and COS-7 cells (ATCC) were maintained in Dulbecco’s modified Eagle’s medium (Lonza) supplemented with 10% fetal bovine serum and 2 mM L-glutamine at 37 °C with 5% CO_2_. Rat pheochromocytoma (PC-12) cells (ATCC) were maintained in DMEM with 5% fetal bovine serum, 5% horse serum, and 2 mM L-glutamine. PC-12 cells used for neurite outgrowth assays were cultured in differentiation medium (DMEM with 1% horse serum, 2 mM L-glutamine, and 100 ng/ml NGF). Cells were transfected using Lipofectamine 2000 (Invitrogen) based on the manufacturer’s instructions. siRNA transfection of PC-12 cells were performed using Lipofectamine RNAiMAX (Invitrogen). P35G-1.5-20-C dishes were purchased from Mattek.

### Confocal imaging

COS-7 cells were fixed in 4% PFA for 20 minutes at room temperature, followed by permeabilization with 0.1% Triton X-100/PBS for 15 minutes. Fixed cells were incubated with primary antibodies for 1 hour at room temperature or overnight at 4 °C, and then secondary antibodies for another hour. All images were taken on an Olympus FV300 confocal microscope or Zeiss LSM700 confocal microscope. For endogenous STIM1 puncta observation, COS-7 cells were treated with 2 μM TG + Ca0 solution for 5 minutes, then fixed in 4% PFA for 20 minutes. The quantification of STIM1 puncta formation was automated by the Find Maxima tool in ImageJ^34^.

### Neurite outgrowth assay

PC-12 cells were dissociated into single cells using trypsin with 0.25% EDTA (Life Technologies). Approximately 1 × 10^5^ cells were seeded on poly-lysine-coated P35G-1.5-20-C dishes and maintained as described above for 6 hours. Differentiation medium was then applied to induce neurite outgrowth for 48 or 72 hours. The images of neurited cells were taken using an Olympus FV300 microscope and the neurite length measured by ImageJ software.

### Single cell [Ca^2+^]_i_ imaging

Ratiometric single cell [Ca^2+^]_i_ imaging was performed on an IX-81 microscope (Olympus)-based system as described previously^48^. HEK293 A cells and COS-7 cells were incubated in DMEM containing 2 μM Fura-2 AM at 37 °C for 45 minutes. PC-12 cells were incubated with 2 μM Fura-2 AM in Ca2 solution for 50 minutes. Data were acquired with Metafluor software (Universal Imaging) and analyzed with OriginPro 8 software (OriginLab) and are expressed as means ± S.E.

### Western Blot

Samples were resolved by SDS-PAGE and analyzed by standard Western blotting. The immunoblot was incubated with the indicated primary antibodies for 1 hour at room temperature or overnight at 4 °C, followed by secondary antibody incubation for 1 hour.

### Statistical Analysis

Data were expressed as means ± SEM. Statistical significance in each group was determined using student’s t test. Significance was determined at a p-value less than 0.05.

## Additional Information

**How to cite this article:** Li, J. *et al*. Atlastin regulates store-operated calcium entry for nerve growth factor-induced neurite outgrowth. *Sci. Rep.* **7**, 43490; doi: 10.1038/srep43490 (2017).

**Publisher's note:** Springer Nature remains neutral with regard to jurisdictional claims in published maps and institutional affiliations.

## Supplementary Material

Supplementary Figures
