# Supplementary material for: Atlastin regulates store-operated calcium entry for nerve growth factor-induced neurite outgrowth
Source: Sci Rep. 2017 Feb 27;7:43490. doi: 10.1038/srep43490 (PMC5327485; doi:10.1038/srep43490)
Supplement: Supplementary Figures [file srep43490-s1.pdf]

Atlastin regulates store-operated calcium entry for nerve growth factor-induced neurite outgrowth

Jing Li[1], Bing Yan[2], Hongjiang Si[3], Xu Peng[3], Shenyuan L. Zhang[3,*], and Junjie Hu[2,*]

[1]Department of Genetics and Cell Biology, College of Life Sciences, Nankai University, and Tianjin Key Laboratory of Protein Sciences, Tianjin 300071, China

[2]National Laboratory of Biomacromolecules, Institute of Biophysics, Chinese Academy of Sciences, Beijing 100101, China

[3]Department of Medical Physiology, College of Medicine, Texas A&M Health Science Center, Temple, Texas, USA

[*]To whom correspondence should be addressed:

huj@ibp.ac.cn (J.H.) and shenyuan.zhang@medicine.tamhsc.edu (S.Z.)

Running title: ER shape affects SOCE and neurite outgrowth

Keywords: endoplasmic reticulum; membrane fusion; Orai; STIM; atlastin; hereditary spastic paraplegia

SUPPLEMENTAL FIGURE LEGENDS

Figure S1. SOCE was down-regulated by overexpression of SPG3A mutants.

(a), Representative confocal images of COS-7 cells transfected with Sec61β-GFP, Sec61β-GFP + Myc-ATL1-Y196C, Sec61β-GFP + Myc-ATL1-R217Q, or Sec61β-GFP + Myc-ATL1-P342S. **(b),** Representative $[Ca^{2+}]_i$ recordings were collected from untransfected COS-7 cells or cells transfected with GFP, GFP + Myc-ATL1-Y196C, GFP + Myc-ATL1-R217Q, or GFP + Myc-ATL1-P342S. **(c),** Averaged peak values of $[Ca^{2+}]_i$ collected from corresponding COS-7 cells (Ctrl, n=47; GFP, n=38; Y196C, n=18; R217Q, n=32; P342S, n=26). Scale bar = 15 μm. *** $P < 0.001$.

Figure S2. SOCE was not changed by overexpression of ER protein Sec61β.

(a), Representative intracellular free calcium ($[Ca^{2+}]_i$) recordings showing TG-triggered SOCE in HEK293A cells transfected with GFP (***left***) or Sec61β-GFP (***right***). **(b),** Averaged peak values of $[Ca^{2+}]_i$ after TG treatment are summarized (GFP, n=49; Sec61β-GFP, n=35). **(c),** Representative $[Ca^{2+}]_i$ data illustrating SOCE after TG treatment in COS-7 cells overexpressing GFP (***left***) or Sec61β-GFP (***right***). **(d),** Averaged peak values of $[Ca^{2+}]_i$ were collected from corresponding COS-7 cells (GFP, n=33; Sec61β-GFP, n=13). **(e),** Representative confocal images of COS-7 cells transfected with Myc-vector, Myc-ATL1-wt, or Myc-ATL1-K80A. The ER morphology was identified by calreticulin antibody (green) and myc antibody (red). Enlarged areas are marked by the small squares, showing normal ER morphology

(left), increased sheet-like structures (***middle***), and a long, unbranched ER tubular network (***right***). **(f),** Representative Western blot data showing the endogenous expression levels of STIM1 and Orai1 in COS-7 cells overexpressing Myc-Vector, Myc-ATL1-wt, or Myc-ATL1-K80A. **(g),** STIM1 puncta were stained in COS-7 cells overexpressing GFP or Sec61β-GFP. **(h** and **i),** Quantification data showing puncta per cell (h) and puncta per 10 μm (i) (GFP, n=24; Sec61β-GFP, n=22). Scale bar = 15 μm. ns, $P \geq 0.05$.

Figure S3. ATL2 and ATL3 knockdown in PC-12 cells showed mildly decreased SOCE.

PC-12 cells were transfected with control non-targeting siRNA ***(black)*** or siRNAs target rat ATL2 and ATL3 ***(red)*** for 72h. **(a),** Representative intracellular free calcium ($[Ca^{2+}]_i$) recordings showed TG-triggered SOCE. **(b),** Averaged peak values of $[Ca^{2+}]_i$ were collected from corresponding PC-12 cells (siCtrl, n=50; sirATL2/3, n=30). **(c),** ATL1, ATL2 and ATL3 expression level were detected by immunoblotting. GAPDH was used as loading control. **(d),** Quantification for the cells with neurites longer than 15 μm (siCtrl, n=296; sirATL2/3, n=210). The percentage was determined from three independent assays. * $P < 0.05$; ns, $P \geq 0.05$.

Figure S4. Overexpression of ATL1 or depletion of ATLs did not change IP3 receptor activity in COS-7 cells

(a), Representative $[Ca^{2+}]_i$ response to 0.5 mM ATP treatment was recorded in COS-7 cells transfected with GFP + Myc ***(left)***, GFP + Myc-ATL1-wt ***(middle)*** or GFP + Myc-ATL1-K80A (***right***). **(b),** Averaged peak values of $[Ca^{2+}]_i$ were collected from panel (a) (Vector, n=23; ATL1-wt, n=11; ATL1-K80A, n=27). **(c),** Representative intracellular free calcium ($[Ca^{2+}]_i$) recordings were collected from COS-7 cells transfected with control non-targeting siRNA ***(left)*** or siRNAs target ATL2 and ATL3 ***(right)*** for 72h. **(d),** Averaged peak values of $[Ca^{2+}]_i$ were collected from panel (c) (siCtrl, n=52; siATL2/3, n=47). ns, $P \geq 0.05$.

Figure S5. TG-evoked SOCE was suppressed by CRAC channel inhibition.

(a-c), Representative $[Ca^{2+}]_i$ data from PC-12 cells showing TG-evoked SOCE was sensitive to CRAC channel blockers: 10 μM BTP2 (b) or 50 μM 2-APB (c). **(d-f),** Representative $[Ca^{2+}]_i$ recordings of TG-triggered SOCE were performed in PC-12 cells transfected with GFP (d), GFP-Orai1-E106A (e), or GFP-Orai1-R91W (f). **(g-i),** Representative $[Ca^{2+}]_i$ data showing SOCE following store depletion in PC-12 cells treated with control siRNA (g), siOrai1 (h) or siSTIM1 (i). **(j),** Averaged $[Ca^{2+}]$ values were obtained at 3 minutes after vehicle control or CRAC channel blocker treatment (Ctrl, n=123; + BTP2, n=40; + 2-APB, n=28). **(k),** Averaged peak values of $[Ca^{2+}]_i$ were collected from corresponding PC-12 cells in panel (d-f) (GFP, n=26; E106A, n=30; R91W, n=28). **(l),** Averaged peak values of $[Ca^{2+}]$ in PC-12 cells transfected with indicated siRNAs (siCtrl, n=70; siOrai1, n=35; siSTIM1, n=25). **(m),** Western Blot showing decreased endogenous STIM1 or Orai1 levels in PC-12 cells

after sirSTIM1 or sirOrai1 treatment for 72 hours. UT, untransfected cells, *** $P < 0.001$.

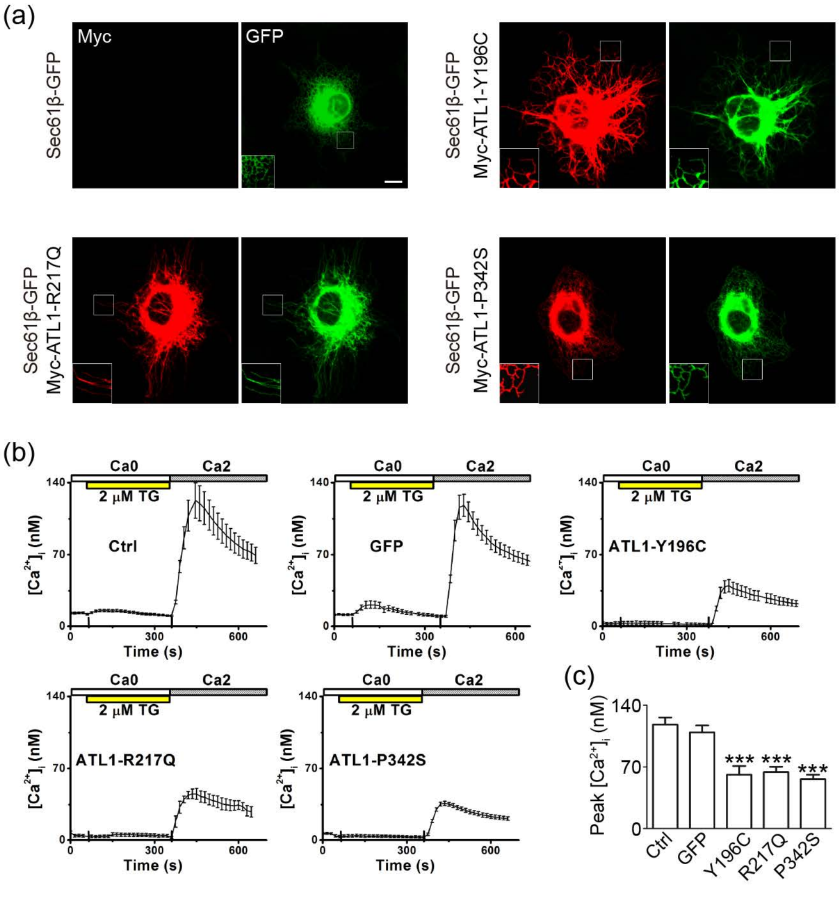

(a)
Myc
GFP
Sec61β-GFP
Sec61β-GFP
Myc-ATL1-Y196C
Sec61β-GFP
Myc-ATL1-R217Q
Sec61β-GFP
Myc-ATL1-P342S
(b)
Ca0
Ca2
2 μM TG
Ctrl
GFP
ATL1-Y196C
ATL1-R217Q
ATL1-P342S
[Ca2+]i (nM)
Time (s)
0
70
140
300
600
(c)
Peak [Ca2+]i (nM)
Ctrl
GFP
Y196C
R217Q
P342S
***

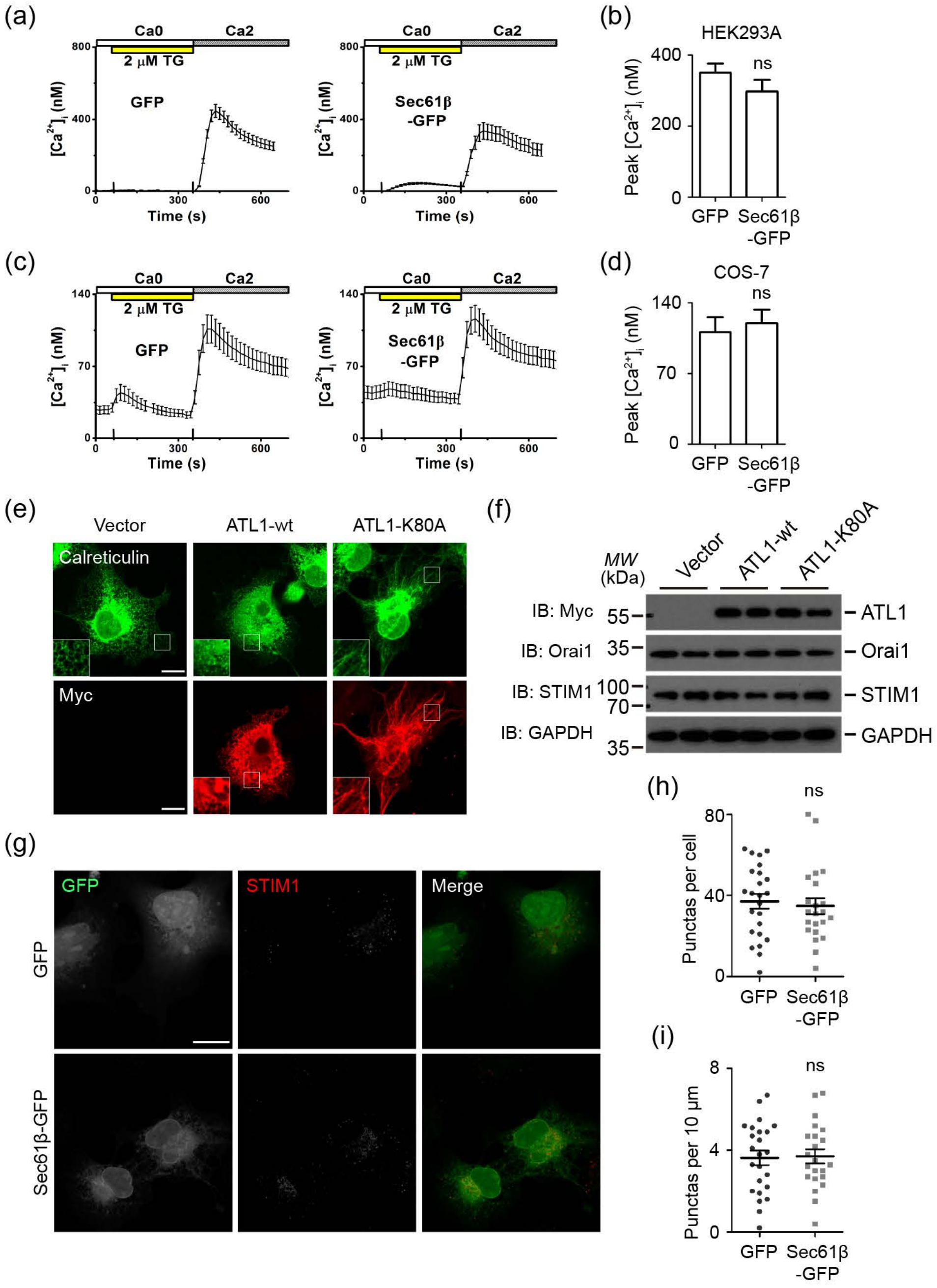

(a)
Ca0
Ca2
2 μM TG
GFP
Sec61β -GFP
[Ca2+]i (nM)
Time (s)
(b)
HEK293A
ns
Peak [Ca2+]i (nM)
GFP
Sec61β -GFP
(c)
(d)
COS-7
(e)
Vector
ATL1-wt
ATL1-K80A
Calreticulin
Myc
(f)
MW (kDa)
IB: Myc
IB: Orai1
IB: STIM1
IB: GAPDH
ATL1
Orai1
STIM1
GAPDH
(g)
GFP
STIM1
Merge
Sec61β-GFP
(h)
Punctas per cell
(i)
Punctas per 10 μm

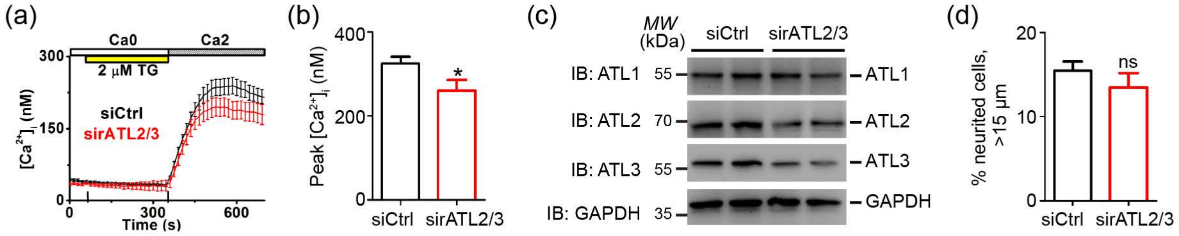

(a)
Ca0
Ca2
2 μM TG
siCtrl
sirATL2/3
[Ca2+]i (nM)
Time (s)
(b)
Peak [Ca2+]i (nM)
siCtrl
sirATL2/3
*
(c)
MW (kDa)
siCtrl
sirATL2/3
IB: ATL1
IB: ATL2
IB: ATL3
IB: GAPDH
ATL1
ATL2
ATL3
GAPDH
(d)
% neurited cells, >15 μm
ns
siCtrl
sirATL2/3

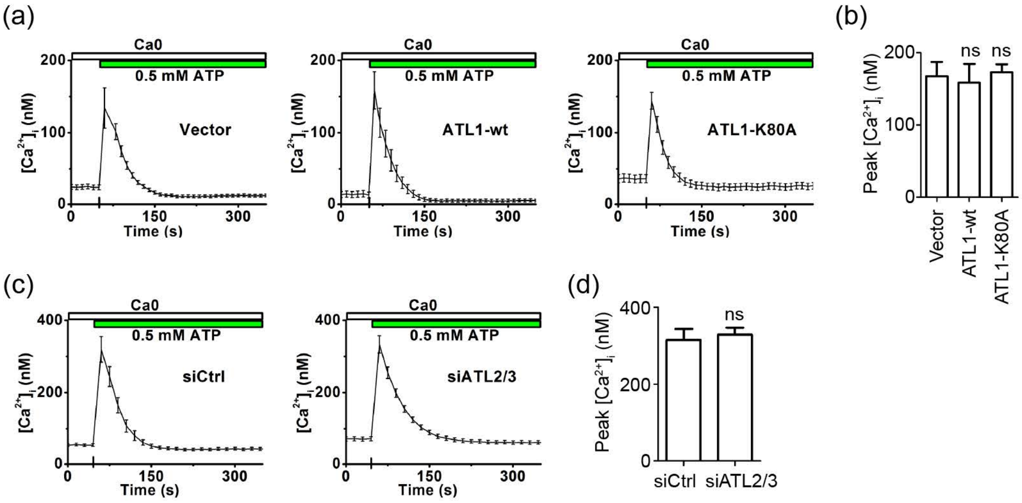

(a)
Ca0
0.5 mM ATP
Vector
ATL1-wt
ATL1-K80A
$[Ca^{2+}]_i$ (nM)
Time (s)
(b)
Peak $[Ca^{2+}]_i$ (nM)
ns
ns
Vector
ATL1-wt
ATL1-K80A
(c)
Ca0
0.5 mM ATP
siCtrl
siATL2/3
$[Ca^{2+}]_i$ (nM)
Time (s)
(d)
Peak $[Ca^{2+}]_i$ (nM)
ns
siCtrl
siATL2/3

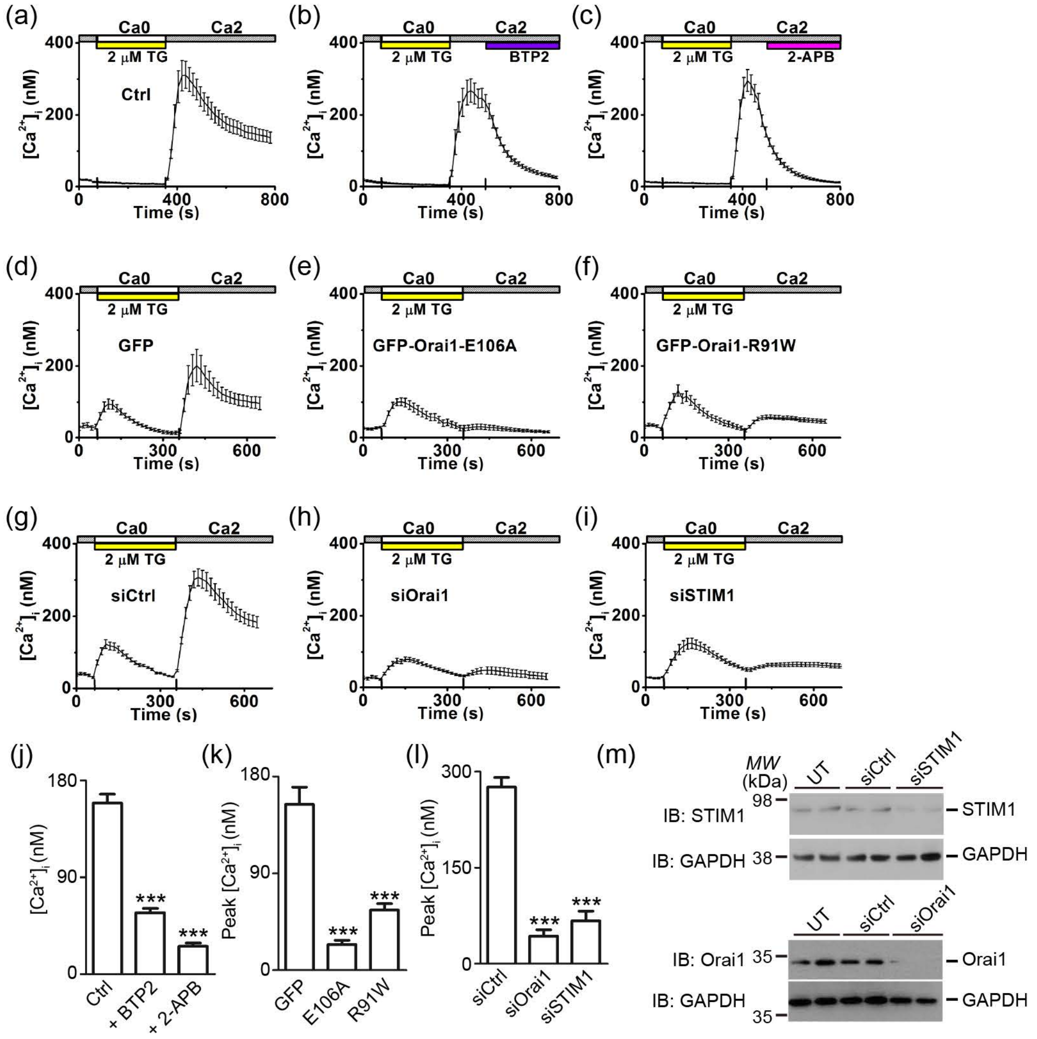

(a)
(b)
(c)
(d)
(e)
(f)
(g)
(h)
(i)
(j)
(k)
(l)
(m)
Ca0
Ca2
2 μM TG
BTP2
2-APB
Ctrl
GFP
GFP-Orai1-E106A
GFP-Orai1-R91W
siCtrl
siOrai1
siSTIM1
[Ca2+]i (nM)
Time (s)
Peak [Ca2+]i (nM)
+ BTP2
+ 2-APB
E106A
R91W
MW (kDa)
UT
IB: STIM1
IB: GAPDH
IB: Orai1
STIM1
GAPDH
Orai1

(a)

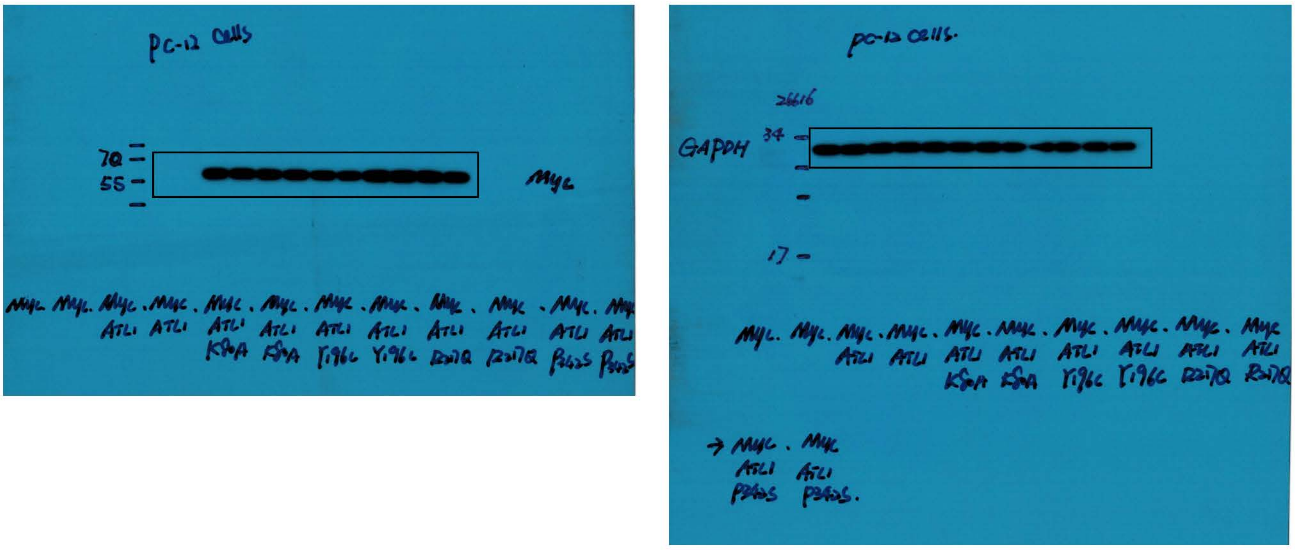
PC-12 cells
70
55
Myc
GAPDH
34
17

(b)

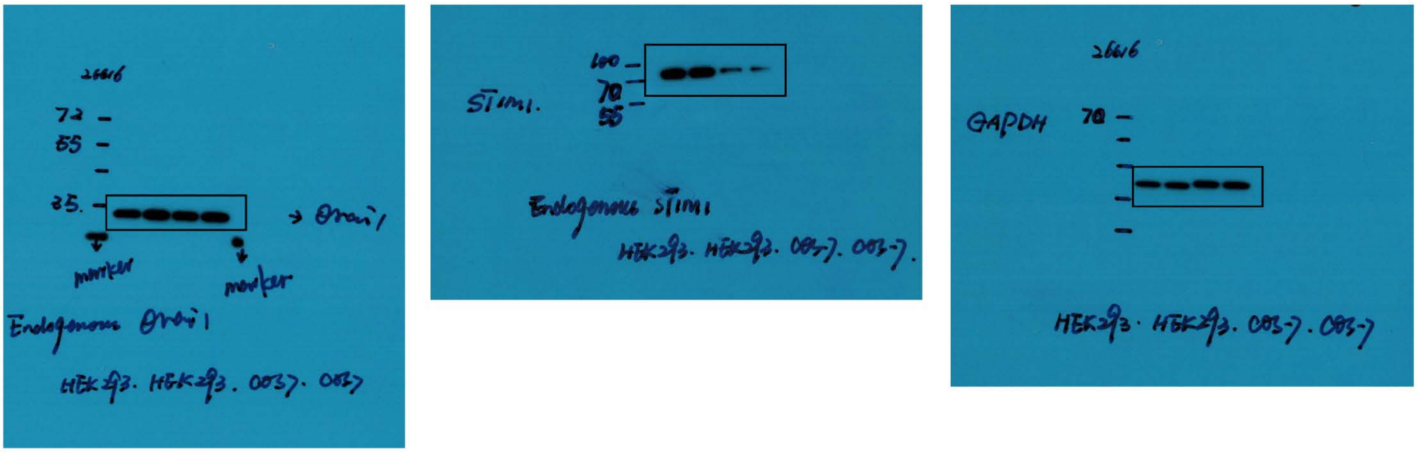
35
marker
marker
STIM1
100
70
55
GAPDH
70

(c)

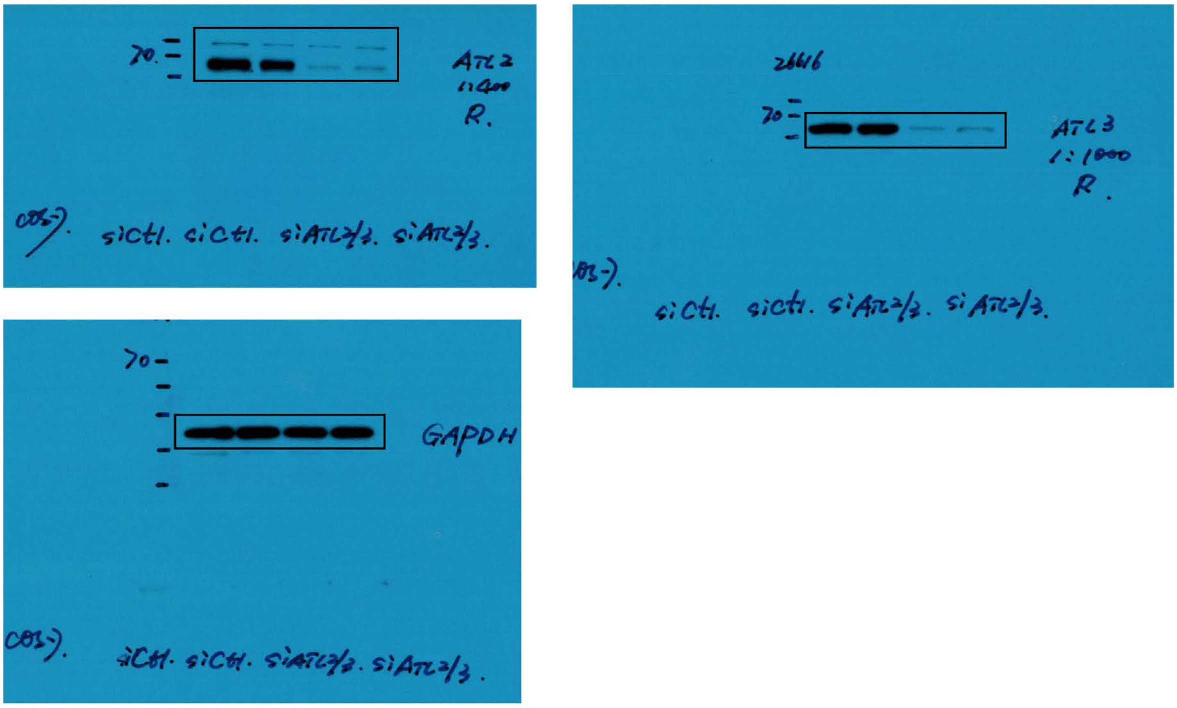
70
ATL2
R.
ATL3
1:1000
R.
GAPDH
